# Effect of mouth rinsing and ingestion of carbohydrate solutions on mood and perceptual responses during exercise

**DOI:** 10.1186/s12970-016-0161-8

**Published:** 2017-01-25

**Authors:** Ajmol Ali, Catherine Moss, Michelle Ji Yeon Yoo, Alanah Wilkinson, Bernhard H. Breier

**Affiliations:** 1 School of Sport and Exercise, Massey University, Albany, Auckland New Zealand; 2 University of Auckland Clinics, University of Auckland, Auckland, New Zealand; 3 School of Applied Sciences, Auckland University of Technology, Auckland, New Zealand; 4 School of Food and Nutrition, Massey University, Auckland, New Zealand

**Keywords:** Perceived exertion, Activation, Affect, Fluid ingestion, Time trial, Sports drink

## Abstract

**Background:**

The aim of this study was to investigate whether mouth rinsing or ingesting carbohydrate (CHO) solutions impact on perceptual responses during exercise.

**Methods:**

Nine moderately trained male cyclists underwent a 90-min glycogen-reducing exercise, and consumed a low CHO meal, prior to completing an overnight fast. A 1-h cycle time trial was performed the following morning. Four trials, each separated by 7 days, were conducted in a randomized, counterbalanced study design: 15% CHO mouth rinse (CHOR), 7.5% CHO ingestion (CHOI), placebo mouth rinse (PLAR) and placebo ingestion (PLAI). Solution volumes (1.5 ml · kg^−1^ ingestion trials and 0.33 ml · kg^−1^ rinsing trials) were provided after every 12.5% of completed exercise. Perceptual scales were used to assess affective valence (feeling scale, FS), arousal (felt arousal scale, FAS), exertion (ratings of perceived exertion, RPE) and mood (profile of mood states, POMS) before, during and immediately after exercise.

**Results:**

There was no difference in RPE (CHOI, 14.0 ± 1.9; CHOR, 14.2 ± 1.7; PLAI, 14.6 ± 1.8; PLAR, 14.6 ± 2.0; *P* = 0.35), FS (CHOI, 0.0 ± 1.7; CHOR, −0.2 ± 1.5; PLAI, −0.8 ± 1.4; PLAR, −0.8 ± 1.6; *P* = 0.15), or FAS (CHOI, 3.6 ± 1.1; CHOR, 3.5 ± 1.0; PLAI, 3.4 ± 1.4; PLAR, 3.3 ± 1.3; *P* = 0.725) scores between trials. While overall POMS score did not appear to differ between trials, the ‘vigour’ subscale indicated that CHOI may facilitate the maintenance of ‘vigour’ scores over time, in comparison to the steady decline witnessed in other trials (*P* = 0.04). There was no difference in time trial performance between trials (CHOI, 65.3 ± 4.8 min; CHOR, 68.4 ± 3.9 min; PLAI, 68.7 ± 5.3 min; PLAR, 68.3 ± 5.2 min; *P* = 0.21) but power output was higher in CHOI (231.0 ± 33.2 W) relative to other trials (221–223.6 W; *P* < 0.01).

**Conclusions:**

In a CHO-reduced state, mouth rinsing with a CHO solution did not impact on perceptual responses during high-intensity exercise in trained cyclists and triathletes. On the other hand CHO ingestion improved perceived ratings of vigour and increased power output during exercise.

## Background

The ergogenic effect of carbohydrate (CHO) ingestion during endurance exercise has been comprehensively documented [1]. However, the focus has been predominantly on performance aspects, meaning less is known about the effect of CHO supplementation on perceptual responses. Studies which do consider such measures, tend to evaluate the relationship using the ratings of perceived exertion (RPE) scale to determine whether CHO ingestion can influence ‘what’ a person feels [2]. The general consensus is that RPE can be attenuated, a phenomenon explained by higher blood glucose levels which permits faster rates of CHO oxidation [3].

In order to gain a broader understanding of individuals’ subjective experiences during exercise, both the feeling scale (FS) and felt arousal scale (FAS) can also be employed [4]. Despite being easily administered during exercise, the use of these tools is a novel area of research, especially where CHO ingestion is concerned [3]. These scales provide single item measures of affect which can supplement findings related to perceived exertion [4]. The FS gauges individuals’ affective valence, specifically ‘how’ a person feels i.e., pleasure or displeasure [5]. Backhouse et al. [6] reported higher FS ratings following CHO ingestion during prolonged cycling demonstrating participants ‘felt better’ in comparison to the placebo ingestion trial, even in the very early stages of the exercise bout. The FAS allows quantification of participants’ perceived level of arousal/activation [7]. Backhouse et al. [6] also found participants to be more activated in the final 30 min of prolonged, high intensity exercise following CHO ingestion. The profile of mood states (POMS) is a common mood evaluation test, providing composite mood scores as well as various subscale scores [8]. Numerous studies have found that CHO ingestion reduced moods such as tension and fatigue, in comparison to ingesting a placebo solution [9, 10].

CHO ingestion before and during exercise is proposed to aid in sustaining optimal functioning of the central nervous system, improving perceptual responses as a result [11]. Duckworth et al. [3] suggest that CHO ingestion prevents the development of a hypoglycaemic state via the maintenance of blood glucose levels. This, in effect, ensures necessary areas of the brain such as the motor cortex remain sufficiently activated [12]. Subsequently, the onset of fatigue is delayed [2]. If hypoglycaemia does develop, this is reported to increase perceived fatigue and lethargy, reduce arousal and undesirably affect mood indicators [3].

The performance improvements ensuing CHO ingestion can also be explained by the maintenance of blood glucose levels [13], in addition to increased CHO oxidation rates [13] and sparing of muscle glycogen [14]. It is important to note, however, that these possible mechanisms apply only to prolonged exercise, such as cycling at a moderate intensity in excess of 2 h [13]. CHO ingestion during exercise of a shorter duration (≤60 min) at a higher intensity has also been shown to improve performance, but for different reasons [15, 16]. Blood glucose reportedly does not decrease and glycogen depletion is not considered to limit exercise capacity [17]. Carter et al. [18] provided evidence to support this notion by testing the effects of intravenous glucose infusion on 1-h cycle time-trial performance. Despite CHO being readily available for oxidation, performance was no different from saline infusion, suggesting that maintaining blood glucose is not imperative for performance in such events [18]. As a result, alternative hypotheses were proposed concerning central as opposed to metabolic mechanisms.

Carter et al. [19] repeated their previous study, adjusting the methodology to involve participants rinsing the CHO solution in their mouths and then expelling it, a technique now recognised as CHO mouth rinsing. Performance improvements were considerably greater when rinsing with CHO compared to placebo, and were equivalent to improvements previously witnessed with CHO [16]. A comparison of both techniques indicated that CHO mouth rinsing may be superior to CHO ingestion where time-trial performance enhancement is concerned [20]. This has been attributed to the activation of oral receptors, stimulating brain centres involved in reward and motivation, ultimately increasing central drive [21]. Researchers have proposed that this phenomenon, also known as the central mechanism hypothesis, may also impact perceptual responses; however evidence to support such claims is limited.

Not all studies show improvements in performance following CHO mouth rinsing. Beelen et al. [22] reported no performance benefits from CHO mouth rinsing when participants were in a fed state. They suggested that, from an evolutionary perspective, it would make more sense for small amounts of CHO in the mouth to be more ergogenic, and affect reward centres in the brain, when glycogen levels were already reduced. However, no studies have examined the effect of CHO mouth rinsing in a fasted state on mood and affect during exercise. Therefore, the aim of this study was to examine whether perceived exertion, affect, activation and mood is influenced by mouth rinsing or ingesting CHO solutions during high-intensity cycling exercise when muscle and liver glycogen stores were compromised. We hypothesised that CHO ingestion and CHO mouth rinse will provide similar perceptual and mood responses during exercise, with both being superior to placebo ingestion and/or rinse.

## Methods

### Participants

This study was part of a larger investigation examining the effects of CHO ingestion and CHO mouth rinsing [23] and the perceptual and mood data will be presented here. Nine male cyclists and triathletes (age 32.7 ± 13.0 y, height 1.80 ± 0.05 m, body mass 72.7 ± 7.3 kg and peak oxygen uptake ($\overset{.}{\mathrm{V}}{\mathrm{O}}_2$ peak) 55.1 ± 7.6 mL · kg^−1^ · min^−1^; mean ± SD) volunteered to participate in this study. They were moderately trained athletes undertaking 5 to 20 h per week of training, interspersed with competitive events. All procedures had prior approval by the Massey University Human Ethics Committee (Southern A 10/01). All participants completed a medical history screening questionnaire and provided written informed consent prior to participation.

### Preliminary measurements

Following anthropometric measurements, participants performed a graded exercise test, on an electronically braked cycle ergometer (model Excalibur, Lode, Groningen, Netherlands), to determine $\overset{.}{\mathrm{V}}{\mathrm{O}}_2$ peak [24] and peak power output (Wmax). They then underwent a short familiarisation of the glycogen reduction exercise protocol [25] before undertaking a full familiarisation of the 1-h cycling time trial protocol [26]. During the familiarisation of the 1-h cycling time trial, the participants were introduced to the perceptual measures and the mouth rinse protocol. They were also provided with information on how to complete the 2-day dietary record.

### Experimental trials

All participants completed four experimental trials, each separated by 7 days, in an air-conditioned laboratory maintained at 18–20 °C. A counterbalanced trial order was used to offset any potential order effects. Participants were asked to avoid consumption of alcohol and caffeine and to record dietary intake over the 2-day period prior to the first main trial and to replicate their intake prior to the other three trials. Their diets were analysed for total energy intake and relative contributions of macronutrients (FoodWorks 5.0, Xyris Software, Australia). The mean energy (13.1 ± 5.7 MJ, 12.7 ± 5.8 MJ, 12.3 ± 5.5 MJ and 11.8 ± 5.4 MJ) and carbohydrate (845 ± 197 g · day^−1^, 694 ± 203 g · day^−1^, 729 ± 195 g · day^−1^ and 705 ± 178 g · day^−1^) intake were not different between trials. Each experimental trial took place over 2 days. On the evening of Day 1, the participants completed a glycogen-reducing exercise protocol followed by a low CHO meal and then a subsequent overnight fast; the following morning participants completed a performance time trial ride.

The glycogen reduction exercise was designed to decrease the glycogen content in both type I and type II muscle fibres [25]. This procedure required participants to cycle for 30 min at 70% $\overset{.}{\mathrm{V}}{\mathrm{O}}_2$ peak, followed by three 50-s ‘sprints’ at double the resistive load (with 2 min rest between each bout), and then a further 45 min at 70% $\overset{.}{\mathrm{V}}{\mathrm{O}}_2$ peak. After completing this exercise participants were provided with a low CHO meal which was the last meal of the day (energy content of 56 kJ · kg^−1^ body mass and CHO content of 1 g · kg^−1^ BM) [26]. Thereafter, they were instructed not to consume any other food but were allowed to consume water ad libitum. Participants arrived in the laboratory the following morning after having fasted for 10–12 h.

Upon arrival on the morning of Day 2 participants’ nude body mass was measured and a cannula was inserted into an antecubital vein (kept patent by frequent flushing with sterile saline). Following a 10-mL resting blood sample and a brief warm-up (5 min at 40% Wmax), participants completed the 1-h time trial [27]. Briefly, participants performed a pre-determined amount of work as fast as possible based on the following:

Total work (J) = 0.75 ∙ Wmax ∙ 3600 s

Where the total amount of work (J) performed was calculated by assuming that the participants could cycle at 75% of their maximum power output (Wmax) for 60 min [27]. Power output during the performance trial was self-selected. The changes of power output were recorded by the investigator and the total amount of work to be performed was recalculated based on the power output at that point in time. Participants received no verbal encouragement and no information of performance other than the amount of work completed.

### Trial solutions

The four trial solutions included A) carbohydrate mouth rinse (CHOR), B) placebo mouth rinse (PLAR), C) carbohydrate ingestion (CHOI) and D) placebo ingestion (PLAI). In Trial A, a 15% CHO solution was used for rinsing and in Trial C a 7.5% CHO solution was used for ingestion. The higher energy content for the mouth rinse solution was chosen as functional magnetic resonance imaging (fMRI) studies have used similar concentrations and shown changes in brain function and improvements in motor output [28]. The placebo solutions (Trials B and D) were taste and colour matched and contained 0% CHO and artificial sweeteners (aspartame). The solutions had a mandarin flavour, were manufactured on-site at the university’s food technology laboratory and were stored in the laboratory food refrigerator (Fisher and Paykell, c450, New Zealand). Trial solution administration and recipes were produced according to previously established methods [29].

During the ingestion trials 1.5 mL∙kg^−1^ body mass solutions were consumed using a sipper bottle. The participant was informed to finish the solution when it was given to them. The trial solution was given every 12.5% of exercise completed. During the mouth rinsing trials participants were required to rinse 0.33 mL∙kg^−1^ body mass solutions (provided in a plastic volumetric syringe; Omnifix 50/60 ml Luer; Germany). Participants self-administered the mouth rinse and were asked to swirl the solution in their mouth for 8 s. After rinsing, participants expectorated all of the solution into a pre-weighed container which was then accurately measured using electronic scales accurate to 0.0001 g (Sartorius LE3235, Germany). The mouth rinse was also administered every 12.5% of exercise completed.

### Perceptual measures

A number of perceptual measures were recorded before, during and after the 1-h time trial. The FAS ranges from 1 indicating low arousal/activation characterised by feeling bored, relaxed and/or calm to 6 which indicates high arousal/activation characterised by feeling angry or excited. The FS is an 11-point scale ranging from -5 (feeling very bad) to +5 (feeling very good) with markers in between these points. The RPE scale [30] ranges from 6 (very, very light) to 20 (very, very hard). These measures were administered every 25% of exercise completed. The shortened POMS [31] is an adjective check list consisting of 37 items rated on a 5-point scale that ranges from ‘not at all’ to ‘extremely’; six factors are derived: tension-anxiety, depression-dejection, anger-hostility, fatigue-inertia, vigour-activity and confusion-bewilderment. The questionnaire was used immediately prior to and immediately following the performance test.

### Blood dispensing and analysis

Blood was collected at rest and after every 25% of exercise completed during the 1-h time trial. The sample was collected in an EDTA tube and centrifuged at 1500 G (Hanil, MF50, Korea) for 10 min at 4 °C and then placed in a −80 °C freezer (Thermaforma 929, Ohio, USA). Plasma glucose was determined using a hexokinase method (Roche Diagnostics, Basel, Switzerland; Flexor E, Vital Scientific NV, 6956 AV Spankeren/Dieren, The Netherlands).

### Statistical analysis

Data were compared using a two-way analysis of variance (ANOVA) with repeated measures (SPSS version 18.0. Chicago, IL) to examine main effects of i) treatment (PLAR, PLAI, CHOR, CHOI) and ii) time (percentage of exercise completed) and iii) interaction of treatment x time. Mauchly’s test for sphericity was applied to the data to examine if sphericity was violated. When sphericity was violated, the Huynh-Feldt estimate was used to correct the data. When significant differences between the interventions were identified by ANOVA, post-hoc Student’s *t*-test, using the Holm-Bonferroni adjustment, was performed. Correlations between variables were examined using simple linear regression equations and reported as Pearson’s correlation coefficient (r). A small (weak) correlation was defined as ± .10 to ± .29, medium (moderate) correlation as ± .30 to ± .49 and large (strong) as ± .50 to ± 1.00 [32]. Data is presented as means ± SD. Statistical significance was set at *P* < 0.05.

## Results

### Perceived exertion (RPE)

**Fig. 1:**
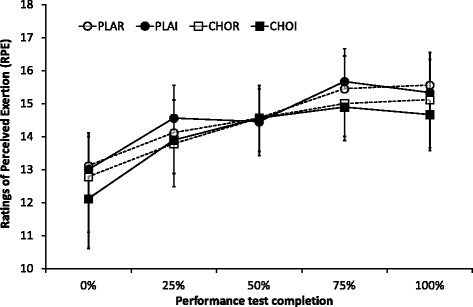
Ratings of perceived exertion (RPE) measured at various intervals throughout the time trial. CHOI – carbohydrate ingestion trial; CHOR – carbohydrate mouth rinse trial; PLAI – placebo ingestion trial; PLAR – placebo mouth rinse trial

RPE increased from 12.8 ± 1.9 at the start of the time trial to 15.2 ± 1.9 at the end of the time trial (main effect of time, *P* = 0.009). There were no effects of treatment (*P* = 0.35) or interaction of treatment x time (*P* = 0.84; Fig. 1).

### Pleasure-displeasure (FS)

**Fig. 2:**
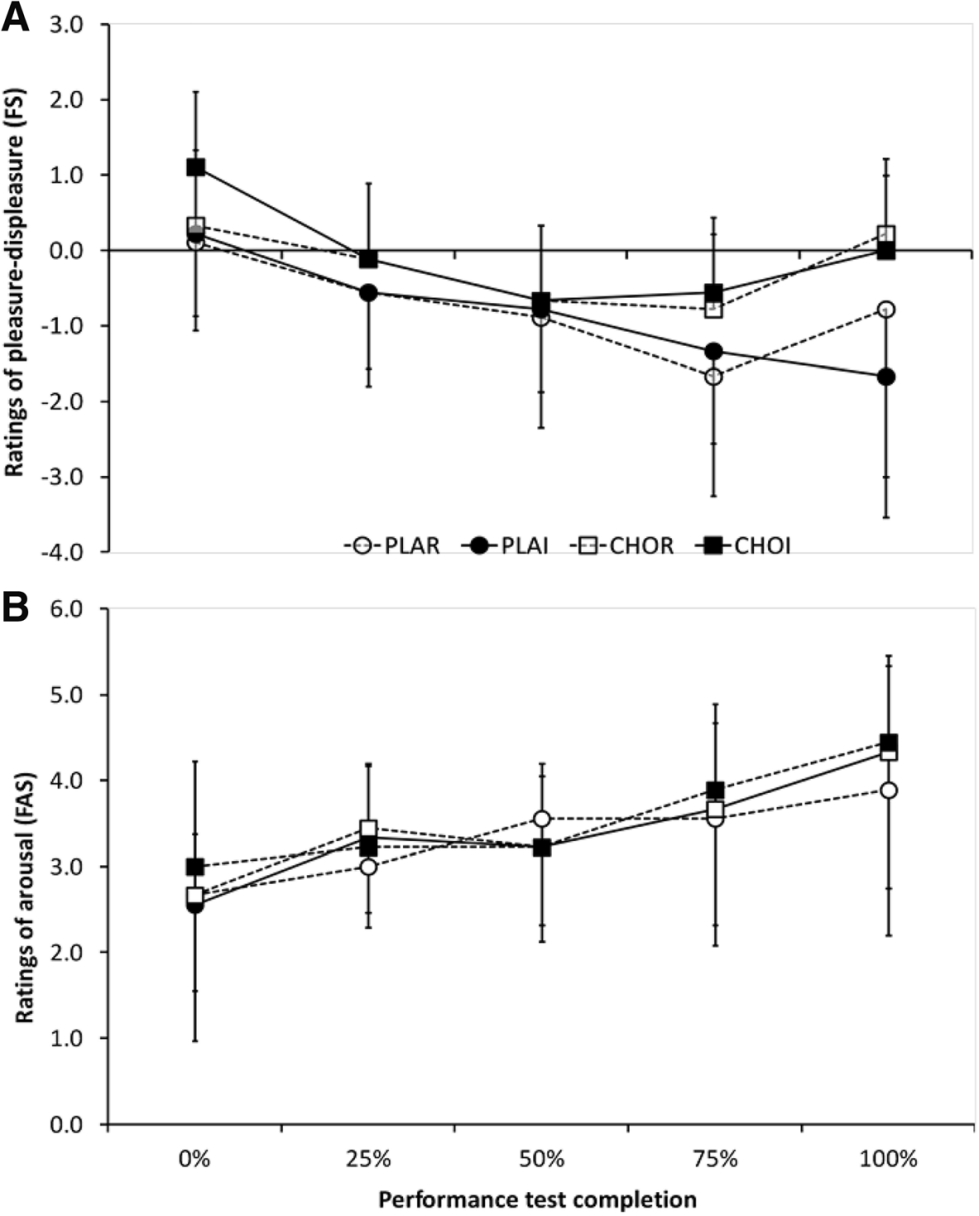
**a** Feeling scale (FS; ‘pleasure-displeasure’) and **b** felt arousal scale (FAS; ‘activation’) ratings during the time trial. CHOI – carbohydrate ingestion trial; CHOR – carbohydrate mouth rinse trial; PLAI – placebo ingestion trial; PLAR – placebo mouth rinse trial

FS ratings decreased with time (*P* = 0.02); mean values at the start of the time trial (0% exercise completed) were higher than all other values (*P* < 0.05). There were no effects of treatment (*P* = 0.15) or interaction of time x treatment (*P* = 0.23; Fig. 2a).

### Perceived activation (FAS)

FAS ratings increased with time (*P* < 0.01); values increased at each time point from the start of the time trial (2.7 ± 1.2) till the completion of the time trial (4.3 ± 1.3; *P* < 0.05 for all pairwise comparisons). There were no effects of treatment (*P* = 0.73) or interaction of treatment x time (*P* = 0.73; Fig. 2b).

### Mood (POMS)

**Fig. 3:**
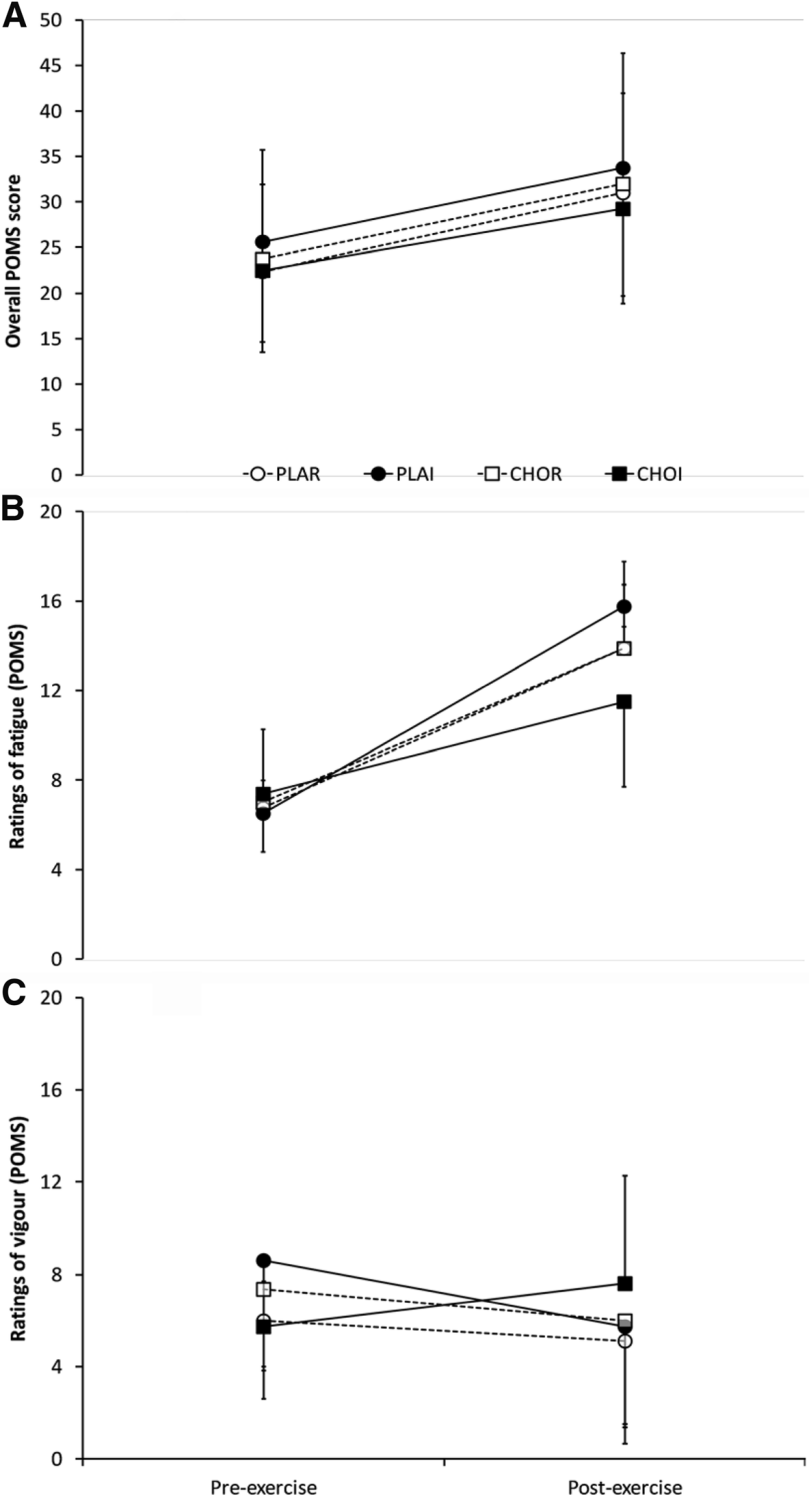
Profile of mood states (POMS); **a** overall (composite) POMS score; **b** fatigue subset ratings only; **c** vigour subset ratings only. CHOI – carbohydrate ingestion trial; CHOR – carbohydrate mouth rinse trial; PLAI – placebo ingestion trial; PLAR – placebo mouth rinse trial

The overall composite POMS score increased from pre to post exercise (23.6 ± 8.5 vs. 31.5 ± 10.9, main effect of time, *P* < 0.01) but there were no effects of treatment (*P* = 0.28) or interaction of treatment x time (*P* = 0.96; Fig. 3a). For the ‘fatigue’ subscale there was a main effect of time with values increasing from 6.9 ± 3.0 to 13.8 ± 4.0 (*P* < 0.01). There was no effect of treatment (*P* = 0.52) for ‘fatigue’ ratings but a trend for an interaction of treatment x time (*P* = 0.08; Fig. 3b). For the ‘vigour’ subscale, there was no effect of time (6.9 ± 3.5 vs. 6.1 ± 4.4, *P* = 0.51) or treatment (*P* = 0.63). There was, however, an interaction of treatment x time (*P* = 0.04) for ‘vigour’ ratings with CHOI values maintained from pre to post-exercise but a concomitant fall in other trials (Fig. 3c).

### Plasma glucose

**Fig. 4:**
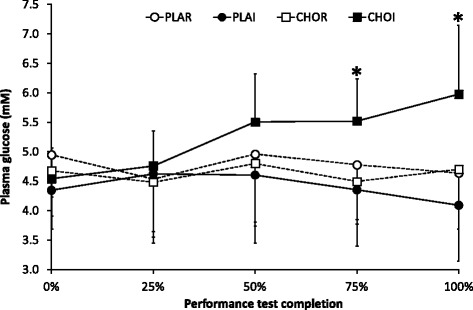
Plasma glucose measured after each 25% of exercise completed. CHOI – carbohydrate ingestion trial; CHOR – carbohydrate mouth rinse trial; PLAI – placebo ingestion trial; PLAR – placebo mouth rinse trial. * indicates significantly higher ratings in CHOI relative to other trials (*P <* 0.05)

There was no effect of time for plasma glucose levels (4.6 ± 0.7 mM (start) vs. 4.9 ± 1.1 mM (end of exercise), *P* = 0.31). However, there was a main effect of treatment (*P* < 0.01) with CHOI (5.3 ± 0.9 mM) higher than all other trials (4.4–4.8 mM, *P* < 0.05). Furthermore, there was an interaction of treatment x time (*P* < 0.01) with CHOI higher than all other trials at 75 and 100% of exercise completed (*P* < 0.05; Fig. 4).

### Exercise performance

Although there were no statistically significant differences in time trial performance between trials (*P* = 0.21) CHOI (65.3 ± 4.8 min) was 4.5–5.2% faster than other trials (CHOR, 68.4 ± 3.9 min; PLAI, 68.7 ± 5.3 min; PLAR, 68.3 ± 5.2 min; Cohen’s d = 0.57–0.65 for pairwise comparisons). There was a decline in power output throughout the time trial for all conditions (main effect of time, *P* < 0.01). However, relative to other trials (221–223 W), power output was higher in CHOI (231 ± 33 W; main effect of treatment *P* < 0.01; Cohen’s d = 0.21–0.31).

## Discussion

The aim of this study was to examine the effects of CHO mouth rinse, placebo mouth rinse, CHO ingestion and placebo ingestion on the perceptual responses of male cyclists exercising in a glycogen-reduced state. As expected RPE and FAS increased whereas FS decreased from the start to the end of exercise but there were no differences between trials. The composite POMS score was not influenced by mouth rinsing or ingestion. However, there was an interaction effect for the ‘vigour’ subscale with higher ratings over time in CHOI relative to the other three trials.

The progressive increase in RPE witnessed in the present study is common in exercise performance trials. Researchers have made a direct link between RPE and performance, suggesting that by attenuating a rise in RPE, performance can be improved [33]. Reducing participants’ perception of exertion when exercising at a given intensity may result in a compensatory increase in exercise intensity, permitting performance improvements otherwise unattainable [33]. RPE values between the four trials in the present study did not appear to differ, however an increase in power output was evident in the trial involving CHOI [23]. Specifically, although there were no differences in performance time between trials (*P* = 0.21), power output was higher in CHOI (231 ± 33 W) relative to other trials (221–224 W; main effect of treatment, *P* < 0.01). Furthermore, power output decreased throughout the time trial (*P* < 0.01) but there was no interaction of treatment x time (*P* = 0.41). This suggests that the ingestion of glucose may have facilitated the ergogenic effect, without the concomitant rise in perceived exertion expected. Chong et al. [34] came to a similar conclusion when comparing the combined effect of ingesting and mouth rinsing with either CHO or placebo solutions. Despite peak power output being higher in the glucose trial, RPE did not differ [34].

This poses the question regarding whether glucose ingestion can facilitate an improvement in peak power output, without causing a concomitant rise in RPE. An attempt to explain such a phenomenon was made by Gant et al. [35] who found that corticomotor output to muscles could be aided by glucose ingestion, whether the muscles were fatigued or not. Silva et al. [36] attribute this outcome to the activation of reward areas of the brain via stimulation of CHO receptors in the mouth. Such brain areas, specifically the insula/frontal operculum, orbitofrontal cortex and striatum, are proposed to be involved with lowering perceived exertion during exercise by supressing fatigue signals sent from the muscles to the brain [4, 20, 21]. While this commonly reported theory seems plausible, it does not provide a justification for why an increase in power output was not witnessed in the CHOR trial [23]. Gant et al. [35] suggest that subsequent increases in force production following CHO ingestion could be attributed to peripheral factors such as the rise in blood glucose, as opposed to central factors involving the primary motor cortex. It is likely that performance improvements witnessed as a result of CHOI are due to the higher oxidation rate, prompted by initially reduced glycogen stores. Commencing experiments with a glycogen reducing exercise was decided based on an initial review of similar literature. Beelen et al. [22] found that CHO mouth rinsing had no ergogenic effect when participants underwent trials in the fed state. In order to determine whether mouth rinsing may have a stronger impact in a CHO-depleted state, participants’ liver and muscle glycogen was diminished through an exercise protocol and an overnight fast the day prior to experimental trials. Despite this, the ergogenic properties of CHOI prevailed over that of mouth rinsing, leading to enhanced vigour, as measured using the POMS questionnaire.

Considering the purported impact of CHO mouth rinsing on areas of the brain associated with reward and pleasure [21] there is little research examining perceptual responses and mood during exercise. Our study is the first to specifically examine perceptual and mood responses following ingestion and rinsing with both CHO and placebo solutions. Rollo et al. [37] reported that mouth rinsing with CHO significantly increased FS ratings (higher pleasurable feelings) immediately prior to the 30-min run in comparison to rinsing with a placebo solution. However, this variance did not remain once the exercise had commenced [37]; this finding is contrary to that of the present study whereby no differences were observed at any stage of the trial between either mouth rinse solutions. Backhouse et al. [6] reported heightened levels of pleasure following CHO ingestion during a longer exercise bout consisting of 120 min cycling. It appeared to attenuate the decline in pleasure ratings which occurred following placebo ingestion, a discovery attributable to higher blood glucose and a reduction in plasma cortisol [6]. This is contrary to findings of the present study, in which no apparent differences between ingesting or rinsing a CHO solution were apparent.

**Fig. 5:**
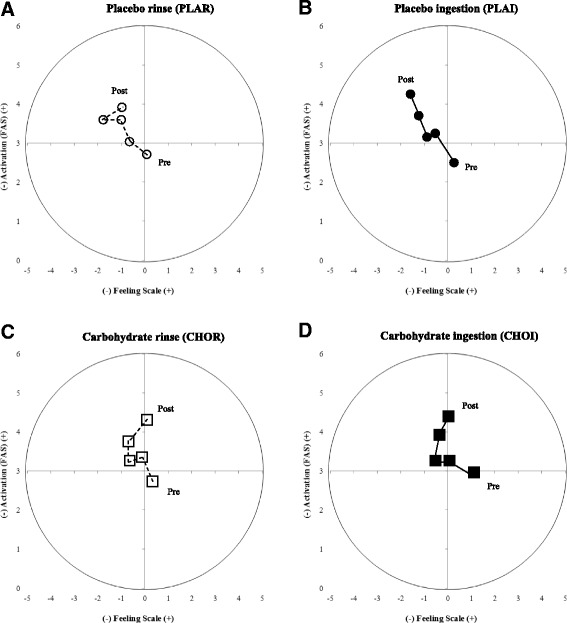
Circumplex model of affect using felt arousal scale (FAS; y-axis) and feeling scale (FS; x-axis) ratings. **a** PLAI – placebo ingestion trial; **b** PLAR – placebo mouth rinse trial; **c** CHOI – carbohydrate ingestion trial; **d** CHOR – carbohydrate mouth rinse trial

A multidimensional approach to perceptual responses during exercise can be achieved simultaneously assessing FS and FAS results using what has been described as the ‘circumplex model’ [38]. In isolation, FS and FAS results may provide insufficient evidence to confidently conclude the effectiveness of a technique, however the combination provides a more in-depth insight into an individual’s subjective experience. Backhouse [39] applied the circumplex model to CHO ingestion, however the application to studies involving CHO mouth rinsing has previously not been explored. A comparison of the circumplex models populated for both the CHO mouth rinsing and CHO ingestion trials from the present study (Fig. 5) provided insufficient evidence to conclude the superiority of one treatment over the other. Pre- and post-trial values were similarly positioned, as were the measured time points in-between. However, comparing CHOR and CHOI to their placebo counterparts revealed noticeable shifts in post-trial value quadrant positions (Fig. 5). Trials involving CHO, regardless of whether this was rinsed or ingested, resulted in post-trial values nearer the ‘high-activation, pleasure’ quadrant, whereas the placebo trials resulted in end values situated in the ‘high-activation, displeasure’ quadrant. This suggests that CHO administration via either ingestion or mouth rinsing may prove beneficial where energy, vigour, revitalisation and excitement are concerned [40]. However, the data is not overly clear therefore the application of the circumplex model to similar future studies would be highly recommended.

Phillips et al. [41] did not ascertain a link between CHO mouth rinsing and sensations of arousal. However they propose that the impact on perceptual measures may be reflected in other central adaptations or alternatively may be due to the fact that the FAS was not sensitive enough. Nevertheless, results from the present study suggest that there were very few alterations to any perceptual measures as a result of the mouth rinsing intervention. Rollo et al. [37] also failed to observe a change in arousal following CHO mouth rinsing. In contrast, others have shown significant improvements in perceived activation when CHO was ingested during prolonged exercise [4, 6]. Such a finding is attributed to the arrival of energy in the form of carbohydrate following exposure to CHO in the mouth, meaning solely rinsing the mouth may subconsciously deceive participants when feedback is sent to the brain that no fuel has arrived [4, 6]. The discrepancy between research by Backhouse et al. and that of our study may be explained by significant differences in protocol, with the 1 h cycling time trial of the present study being of a much shorter duration than the 1.5–2 h of cycling completed in both previous studies.

It is also worth noting the relevance of plasma glucose values when discussing perceptual responses. In the present study, despite plasma glucose being significantly higher in the CHOI trials, minimal differences appeared to exist when comparing the subjective experiences of participants between this and the CHOR trials. However, it poses the question of whether the higher work output which CHO ingestion permitted [23], actually negated the possibility of participants’ simultaneously feeling better and more aroused. Such a notion can only be confirmed by adjusting the protocol of a future study, keeping exercise intensity constant to ensure the individuals subjective experience is not confounded by other outcomes.

This is the first study to use the POMS to gauge the effects of CHO mouth rinsing and ingestion in the fasted state meaning direct comparisons to other work is not possible. The suggestion that CHO mouth rinsing can stimulate pleasure and reward centres in the brain implies that mood may be enhanced accordingly. The anticipated effect of CHOR enhancing mood to a greater extent due to the prolonged period for oral receptor activation was not witnessed. This suggests that the speculated areas of the brain involved in this mechanism may in fact not be associated with mood [42]. Despite this observation, the application of the composite POMS score to the present study may in fact not be overly applicable given the context. The composite score considers a variety of mood states, some of which have less relevance where exercise is concerned, for example depression and anxiety. Therefore, the analysis of certain subscales may provide particularly valuable insight. CHOI facilitated the maintenance of ‘vigour’ subscale values from pre to post-exercise (and a trend for reduced ‘fatigue’ ratings), preventing the decline witnessed in all other trials. Winnick et al. [43] propose that the decline in vigour ratings among other trials may be linked to the accumulation of serotonin (5-HT) in the brain by its precursor, tryptophan, ultimately triggered by higher blood free fatty acid (FFA) concentrations and consequential tryptophan transport to the brain. 5-HT is reported as having dopamine-inhibitory effects, a consequence which appears to be prevented by CHOI [43]. However, without measuring 5-HT and tryptophan levels such explanations can only be regarded as speculation.

Although the combined application of RPE, FS, FAS and POMS enables an all-encompassing evaluation of perceptual measures, future studies in this area would benefit from increasing participant numbers due to the sensitivity of these measures. A further limitation was the inability to blind subjects to which protocol was taking place due to the nature of this experiment.

## Conclusions

Mouth rinsing with CHO did not influence participants’ perceived exertion, pleasure or perceived activation before, during or after a 1 h cycle time trial relative to placebo. However, the ingestion of CHO did facilitate increases in plasma glucose and performance improvements as measured by power output. The improvement in power output evident when participants ingested a CHO solution suggests that consumption may aid in preventing the concomitant rise in perceived exertion generally seen when a greater amount of work is completed. Similarly, overall mood assessed via the POMS questionnaire did not appear to be influenced by either mouth rinsing or ingestion of CHO when compared to placebo. Interestingly, CHO ingestion did seem to prevent the typical decline in vigour ratings from pre to post-exercise seen in the remaining three trials. A link has been proposed between this finding and dopamine inhibitory effects; however more research is warranted to confirm or refute this suggestion. It is possible that in extreme CHO-reduced conditions, such as those faced by the participants in the present study, potential activation of cranial pathways by mouth rinsing is not enough to offset the necessity for plasma glucose levels to be maintained.
